# Nanofiltration membrane for bio‐separation: Process‐oriented materials innovation

**DOI:** 10.1002/elsc.202000100

**Published:** 2021-03-20

**Authors:** Yang Cao, Guoqiang Chen, Yinhua Wan, Jianquan Luo

**Affiliations:** ^1^ State Key Laboratory of Biochemical Engineering Institute of Process Engineering Chinese Academy of Sciences Beijing P. R. China; ^2^ School of Chemical Engineering University of Chinese Academy of Sciences Beijing P. R. China

**Keywords:** interfacial polymerization, membrane fouling, membrane separation, nanomaterials, polymeric membranes

## Abstract

Nanofiltration (NF) with advantages of high efficiency and low‐cost has attracted increasing attentions in bio‐separation. However, the large‐scale application is limited by the inferior molecular selectivity, low chemical stability and serious membrane fouling. Many efforts, thus, have been devoted in NF materials design for specific applications to enhance the separation efficiency of bio‐products and increase membrane life‐time, as well as reduce the operating cost. This review summarized the recent progress of NF applications in bio‐separation, discussed various demands for NF membrane in the bio‐products purification and corresponding material innovations, finally proposed several practical suggestions for future research, which provided directions and guidance toward further product development and process industrialization.

**Abbreviations**
CC: cyanuric chloride
COF: covalent‐organic framework
GO: graphene oxide
IP: interfacial polymerization
MOF: metal‐organic framework
MoS_2_: molybdenum disulfide
MPD: m‐phenylenediamine
MWCO: molecular weight cut‐off
NF: nanofiltration
PA: polyamide
PBI: polybenzimidazole
PEEK: poly(ether ether ketone)
PEI: polyethyleneimine
PEI‐g‐SBMA: polyethylenimine‐sulfobetaine methacrylate
PES: poly(ether sulfone)
PI: polyimide
PIP: piperazine
PPESK: poly(phthalazine ether sulfone ketone)
SRNF: solvent‐resistant nanofiltration
TFC: thin film composite
TMC: trimesoyl chloride
UF: ultrafiltration

## 1 INTRODUCTION

**FIGURE 1:**
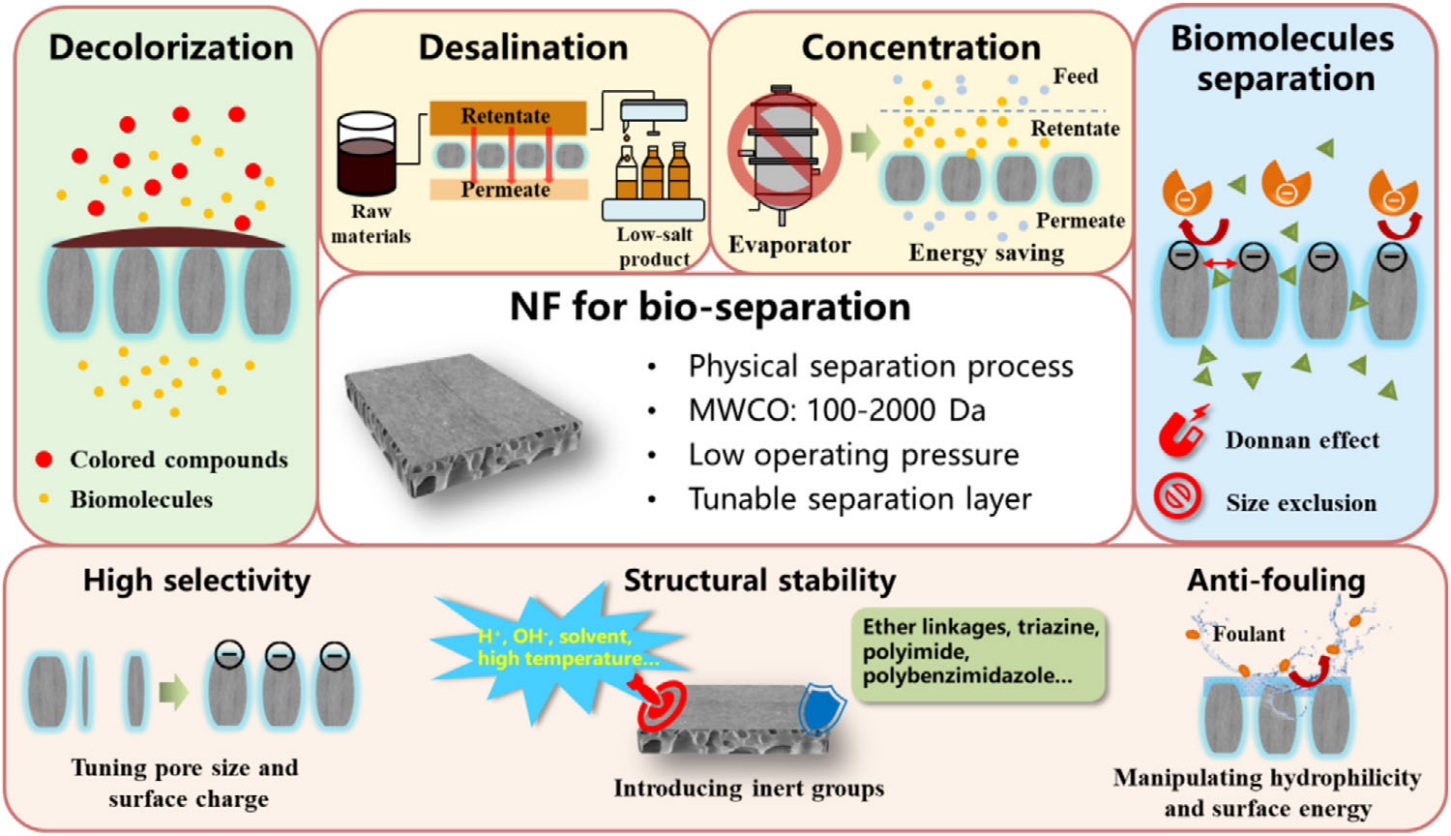
Applications of NF membrane for bio‐separation and schematic of different material innovations covered in this review

Recent years have witnessed tremendous advance of life science and bio‐product manufacturing, which spawned great requirements for cost‐effective purification and recovery technologies to ensure products with high purities and bioactivities [1]. However, conventional purification strategies, such as activated carbon adsorption, resin based ion exchange and column chromatography are semi‐continuous processes with massive solid waste generation and product loss, as well as high pretreatment/regeneration costs [2]. Membrane technology, especially nanofiltration (NF), as an energy‐saving and environmentally friendly separation technology, plays more and more important role in purification and recovery of the high‐value bio‐products [3]. NF membranes with the properties including pore size <2 nm, molecular weight cut‐off (MWCO) of 100–2000 Da and generally charged surface in aqueous environments, have drawn particular attention due to their predominance in selectively rejecting biomolecules. As a result of these features, NF membranes are suitable for various applications such as decolorization, desalination, concentration and biomolecules separation (Figure 1) [4]. Compared to conventional purification strategies, the greatest advantage of NF is the ability to recycle valuable substances (e.g. pigments, inorganic salts and water) without generating new contaminants. In addition, compared to evaporation concentration processes, NF can achieve the effective removal of impurities with less than 20% of the energy consumption and avoid massive chemical reagents consumption [5]. Especially for the biomolecules with similar molecular weight, the pore structure and surface charge pattern of NF membrane can be designed according to the difference of the solutes in size, shape and charge, so as to improve the separation selectivity.

**PRACTICAL APPLICATION**

Membrane technology, especially nanofiltration (NF), as an energy‐saving and environmentally friendly separation technology, plays an important role in bio‐separation. NF membranes with a pore size of 0.4–2 nm and generally charged separation layer are suitable for various bio‐separation applications such as decolorization, desalination, concentration and biomolecules separation. However, the large‐scale applications of NF are still limited in this area due to the inferior molecular selectivity, low chemical stability and serious membrane fouling when facing the complex industrial liquids (e.g. extract/hydrolysate/fermentation broth). Via materials innovation, we attempted to prepare NF membranes with narrow pore size distribution (high separation selectivity), strong anti‐fouling surface as well as good thermal/chemical stability, which can solve most problems occurring during NF process. Therefore, this review summarized the recent progress of NF applications in bio‐separation, discussed various demands for NF membrane in the bio‐products purification and corresponding material innovations, finally proposed several practical suggestions for future research.

Although NF membrane upgrades the products’ bioactivities and simplifies the purification processes, the complicated compositions of extract/hydrolysate/fermentation broth (e.g. salts, proteins, pigments, peptides and saccharides), harsh operating and membrane cleaning conditions make NF applications still suffer the following limitations: first, the insufficient selectivity of biomolecules and small solutes (e.g. salts and pigments) by most commercial NF membranes normally leads to low bio‐product quality and high membrane fouling potential; second, severe membrane fouling would lead to a sharp permeability decline and a variation of separation selectivity [6]; third, high salt/acid/base content in feedstocks, high operating temperature and frequent chemical cleaning greatly shorten membrane life‐time.

The properties of NF membranes are mainly determined by the separation layer materials. Although unflagging efforts have been made over the past few years to improve the NF membrane performances via materials innovation to meet the particular separation requirements, the systematical review on NF for bio‐separation has not been reported. Therefore, this review presents an overview on the applications of NF membrane in bio‐separation, including decolorization, desalination, biomolecules separation and concentration. Then, recent advances in the innovation of NF membrane materials for bio‐separation are introduced. Finally, perspectives are emphasized on the potential opportunities in future research. This review intends to better understand the special requirements of NF membrane for bio‐separation, and also to point out the prospects and future research directions of NF for bio‐separation.

## 2 APPLICATION OF NF IN BIO‐SEPARATION

### 2.1 Decolorization of bio‐products

The undesirable coloring of bio‐products is caused by nature pigments (e.g. flavonoids, chlorophylls, xanthophylls and phenols and anthocyanins) originally existed in biomass, and/or side‐reaction compounds generated during the processing cycle [7]. In order to obtain high‐quality bio‐products, decolorization is the key step after clarification by membrane filtration or centrifugation. As a physical separation method without chemical addition, NF is promising for bioproducts decolorization and nature pigments recovery.

Sugar production is a representative decolorization process that pigments span over a wide range of sizes with low molecular weights [8]. For instance, Gyura et al. obtained a maximum color removal of 76% when using a NF membrane with a MWCO of 500 Da to treat green syrup from sugar beet (intermediate product in sucrose crystallization) [9]. Although the color removal can be further increased by using the membrane with smaller pore size, some performance limitations (i.e. low permeate flux, high sucrose loss in the concentrate and serious membrane fouling) imped the application of NF membrane for decolorization. To achieve high color removal efficiency, Guo et al. systematically evaluated eleven ultrafiltration (UF) and NF membranes for molasses decolorization, and concluded that the hydrophilic and negatively charged polyamide (PA) NF membrane of 300–500 Da exhibited desirable separation selectivity of pigments/sucrose, outstanding permeate flux and anti‐fouling performance [10]. In addition, the integration of NF membrane with other separation technologies was also used to reduce the membrane fouling and recover pigments. Luo et al. obtained a color removal up to 96.6% of sugarcane juice by an integrated membrane process (i.e. clarification, decolorization and concentration by three membranes), where the UF clarification step effectively decreased the membrane fouling during the decolorization step by a loose NF (also called tight UF) [11]. On the other hand, to recover the valuable substance from cane molasses, an integrated resin adsorption and NF membrane process was proposed by Luo et al., where the extraction of hydrophobic caramel pigments by nonpolar resin could reduce the formation of membrane fouling, and thus, the NF permeate flux during the decolorization process increased by 1.5 times [2]. Finally, polyphenol pigments in the NF retentate could be further purified by diafiltration to remove the residual sugar.

Another common application of NF is beverage decolorization, where colored substances in raw juice need to be removed to improve the product sensory quality. Beverage decolorization by NF is proposed as an alternative to activated carbon adsorption and thermal treatment because it can better keep the bioactive nutrients in juice. Arend et al. found that a NF membrane with a MWCO of 150–300 Da could remove 91% color of strawberry juice without high temperature treatment [12]. Moreover, the NF based decolorization has also been attempted for the purification of various bio‐products and the treatments of their wastewaters, such as baker's yeast, alcohol, succinic acid, silage juice, d‐lactic acid, and soy sauce [7].

### 2.2 Desalination of bio‐products

Many bio‐product manufacturing processes, especially pharmaceutical synthesis, food and fermentation downstream processing, generate raw liquid or wastewater with highly concentrated salts (up to 20%, w/v) due to acid‐base neutralization or salt addition/generation [4, 13]. The excessive salt concentrations in these raw liquids would induce many problems, such as reducing product quality, impairing human cardiovascular health and accelerating equipment scaling, and thus most salt ions should be removed [14]. NF membranes enable to separate monovalent salts and small organic compounds, which is highly recommended for bio‐product desalination. For instance, Luo et al. applied four commercial NF membranes for the desalination of raw soy sauce, where 55% of NaCl was removed but the nutrient substances such as amino acids could be largely retained (>95%) by the NF270 membrane [15]. However, high salt concentration would lead to pore swelling and solute dehydration due to salting‐out effect as well as cause serious charge screening effect during NF process; thus, accelerating the transfer of organic solutes through the membrane. Moreover, concentration polarization of the rejected solutes (e.g. iron dextran) would be aggravated at higher salt concentration and permeate flux, which led to larger osmotic pressure across the membrane and higher filtration resistance on the membrane, resulting in high energy consumption [3, 16]. Therefore, NF was often applied with dilution or diafiltration operations to decrease salt and other solute concentrations. By increasing the ratio of diafiltration water and permeate volumes from 0.5 to 0.75 (variable volume diafiltration), Román et al. found that the removal of monovalent ions in acid whey rose from 70 to 90%, and the rejection of the valuable ingredients like protein and lactose was over 90% [17]. Furthermore, by comparing the desalination of soy sauce by NF in laboratory and pilot‐plant tests, Luo et al. concluded that dilution‐concentration‐diafiltration mode was the most suitable one at laboratory scale due to its shorter processing time and less water consumption, but in the real application, dilution‐concentration mode, also called variable volume diafiltration was preferred attributed to the higher permeate flux and easier operation [15].

### 2.3 Concentration of bio‐products

Since the content of bioactive substances (e.g. sugar, polyphenol, amino acid and protein) in the raw materials is normally low, it is necessary to increase the concentration of these high‐value components and even to obtain their powder/crystal in the final product. Vacuum freeze drying, spray drying and multi‐effect evaporation are the widely used methods to concentrate the bio‐products, but they are also energy intensive processes due to the existence of phase transition. Pre‐concentration of bio‐products by NF can greatly reduce the total energy consumption because it can remove a large amount of water at ambient temperature, which has been widely applied in beverage processing. For example, Warczok et al. reported the concentration of pear and apple juice by NF and the fructose concentration in the juice increased significantly [18]. Moreover, the permeation of the impurities with small molecular weight such as salts, organic acids and monosaccharides can lower the osmotic pressure in the concentrate, which is beneficial to enhance the permeate flux or reduce operating pressure. The higher concentration of bio‐products also favors their bioactive and storage stability. Another typical case is the concentration of cane juice for sugar production, where sucrose needs to be pre‐concentrated for subsequent evaporation and crystallization. Malmali et al. found that the NF270 showed high permeate flux, high sucrose rejection and great permeation of impurities [19]. Luo et al. reported that operating at high temperature, low permeate flux and high sugar content was more favorable for concentrating sucrose and at the same time permeating reducing sugar. However, the flux decline with increase of concentration factor due to fouling formation and exorbitant osmotic pressure is still the main challenge of the NF concentration process, and thus NF can only be used for pre‐concentration step (soluble solids in the concentrate was normally less than 30%) [20].

### 2.4 Separation of biomolecules

NF membrane with tunable pore size and charge pattern is preferable in the separation of small biomolecules such as amino acids, peptides, antibiotics, lactic acid and oligosaccharides. By regulating the pore size and its homogeneity of NF membrane, the precise control of molecular weight of bio‐products can also be achieved [21]. For instance, the NF membranes with loose separation layer enabled to fractionate oligosaccharide and low‐molecular‐weight sugars/other substances, and the large membrane pore size enhanced permeate flux and reduced membrane fouling [22]. Moreover, as many biomolecules are ionizable, Donnan effect plays an important role in their separation. Therefore, pH and ionic strength of the feed solutions, which affect the surface charge properties of solutes and NF membrane, have significant influence on the separation performance. For example, Garem et al. found that a low pH was beneficial to reject basic amino acids via electrostatic repulsion, whereas neutral and acidic amino acids could easily pass through the membrane [23]. By comparing the separation behavior of amino acids by four NF membranes, Ray et al. claimed that the NF membrane with highest negative Zeta potential exhibited significant differences in amino acid rejections at different pH, which might be suitable for the separation of amino acids from their mixture [24]. Using the NTR7450 membrane whose pore size is larger than the size of l‐glutamine, Li et al. evaluated the separation performance of l‐glutamine and glutamate, and the membrane showed a high glutamate rejection of over 90% but 85% passage of l‐glutamine [25]. Moreover, for the separation of sugars with similar structure, size and charge (e.g. glucose and xylose), Morthensen et al. converted glucose to gluconic acid via an enzymatic process, followed by separation of xylose from gluconic acid by NF. The stronger charge property difference between xylose and gluconic acid increased the xylose separation factor from 1.4 to 32 at the same process conditions [26].

## 3 HIGH‐PERFORMANCE NF MEMBRANES FOR BIO‐SEPARATION

Although the applications of NF membrane in bio‐separation have been extensively investigated and reported in literature, its large‐scale industrial applications are rare due to insufficient separation selectivity for small molecules, serious membrane fouling and unsatisfied chemical/mechanical/thermal stability when facing the complex industrial liquids and operated for a long‐term. Though process optimization and intensification may alleviate the above problems, they can be better solved by membrane materials innovation.

### 3.1 Highly selective NF membranes

**FIGURE 2:**
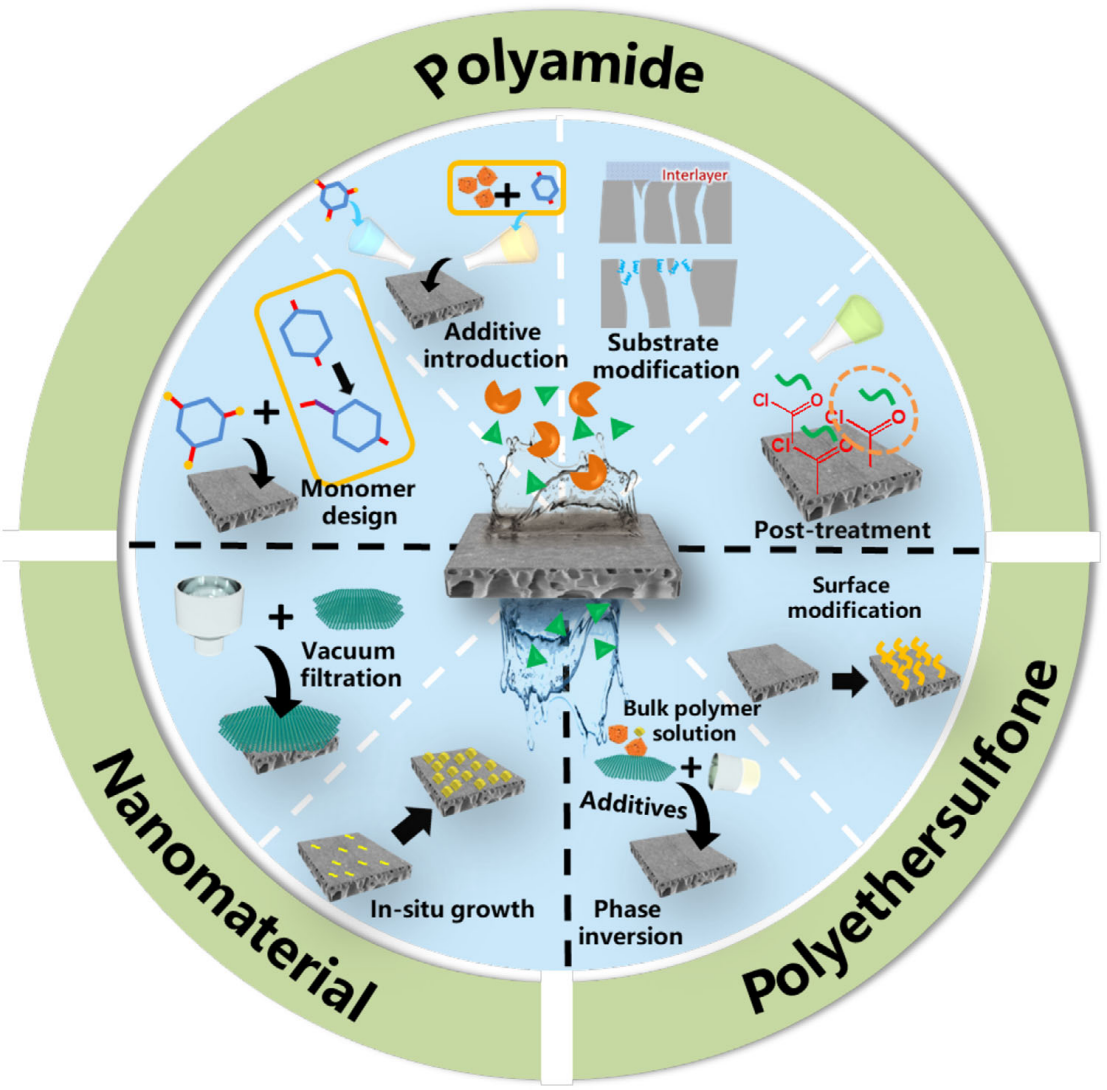
Overview of different methods for highly selective membranes based on PA, PES and nanomaterial separation layers, respectively

Separation selectivity of NF membranes is mainly governed by size exclusion and electrostatic interactions. However, for the most commonly used materials poly(ether sulfone) (PES) and polyamide (PA), the uneven pore size distribution greatly deceases the separation selectivity because the target molecules which are supposed to be retained can readily pass through the membrane via the large pores. Narrowing the pore size distribution, therefore, is an effective way to improve the solute separation selectivity. Moreover, the regulation of membrane surface charge can also lead to an enhanced selectivity of charged biomolecules by virtue of Donnan effect. Highly selective NF membranes with different materials and their separation performances were summarized according to various modification methods as shown in Figure 2 and Table S1, respectively.

**FIGURE 3:**
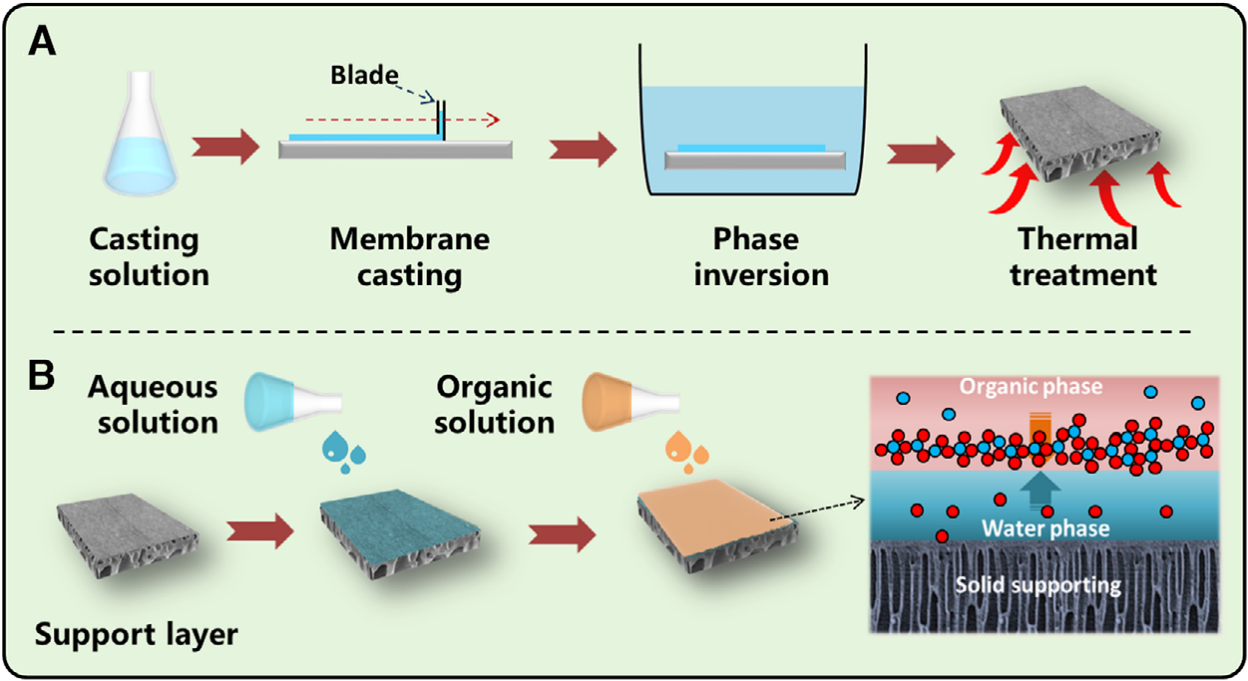
Schematic diagrams of (A) phase inversion and (B) interfacial polymerization processes for membrane preparation

The membranes prepared via the phase inversion method own an asymmetric and loose structure as well as a high salts permeation (Figure 3A), and PES membrane is the most common one. However, its wide pore size distribution and hydrophobic surface are unfavorable for the precise separation [27]. Therefore, nanomaterial blending and surface modification have been applied to tune the pore structure of the PES membrane and thus, improve its separation selectivity. Nanomaterials with high porosity and strong solvent affinity such as graphene oxide (GO), metal‐organic framework (MOF), covalent‐organic framework (COF), molybdenum disulfide (MoS_2_) and MXene have been applied to reduce the thermodynamic stability and enhance the viscosity of casting solution; thus, obtaining PES membrane with thinner separation layer, narrower pore size distribution and more free volume [7]. Meanwhile, hydrophilic nanomaterials in polymer would reach the membrane surface through surface segregation, which is able to improve the hydrophilicity and tune surface charge for higher biomolecule rejection and better salt permeation [28]. However, there are still some limitations for its large‐scale applications including high nanomaterials cost, obvious nanomaterials aggregation during membrane preparation and nonnegligible nanomaterials leakage during filtration. On the other hand, surface post‐treatment can controllably tune the surface pore size, charge, and hydrophilicity by increasing crosslinking degree or introducing additional surface layers; thus, enhancing the separation selectivity and even transforming UF membrane to an NF one [29]. For example, by controlling the release of the nanocapsule decorated polyethyleneimine (PEI) and carbon dioxide, Xia et al. prepared a NF membrane with tunable pore size and charge properties. The result showed that the enhanced molecular sieving, shape‐selectivity and charge exclusion improved the rejection of Janus green B up to 98.7% [30].

PA is the most widely used material for NF membrane due to the good hydrophilicity, mechanical stability, and facile fabrication process. Interfacial polymerization (IP) is commonly applied to prepare thin film composite (TFC) PA membrane on a UF substrate (Figure 3B) [31]. However, uneven surface porosity of the UF substrate and strong reactivity of monomers usually lead to rough surface structure and wide pore size distribution [32]. To solve these obstacles, strategies such as substrate modification, new monomer design, additive introduction and surface post‐treatment are attempted. For the UF substrate, incorporating hydrophilic or amphiphilic molecules can not only increase the surface porosity but also accelerate the diffusion rate of aqueous monomer into the interface; thus, obtaining membrane with low thickness, high permeability and better separation selectivity [33]. Moreover, construction of interlayer/sacrificial layer (e.g. nanoparticle, polyelectrolyte and mussel‐inspired coating) on the substrate can also effectively regulate the surface pore size and hydrophilicity; thus, facilitating the uniform IP process [34]. Using polydopamine as an interlayer, Yang et al. prepared a PA membrane with improved hydrophilicity, increased crosslinking degree and narrower pore distribution, which showed a high permeability (14.8 Lm^–2^h^–1^ bar^–1^) and a desirable antibiotic/monovalent ion selectivity [35]. While for IP process, monomers with high reactivity, low solubility in reverse phase and large size are likely to create more free volume and limit the excessive penetration of monomers into another phase, thereby fabricating uniform PA membrane with high permeability and selectivity [32]. Moreover, by changing solution properties (e.g. pH, viscosity and surface tension) via adding additives such as acid acceptor, co‐reactants, surfactant, nanomaterial and co‐solvent, the mass transfer and reactivity of monomers at the interface could also be regulated. For example, Liang et al. discovered that the commonly used surfactant could not only improve the interface wettability, but also control the monomer transfer rate and the hydrolysis rate of the nascent PA layer, resulting in a narrow pore size distribution and higher selectivity for sugars (sucrose and raffinose) and Na^+^ [36]. Besides, the residual acyl chloride groups on the nascent PA layer can be easily tuned by various post‐treatments such as thermal treatment, solvent activation and second IP. For instance, Guo et al. prepared loose NF PA membrane via a post‐treatment method using different reagents (e.g. ethanol, n‐hexane, ionic liquid), which showed excellent decolorization performance for molasses as well as good selectivity for antibiotics (tobramycin and clindamycin) and NaCl [37].

It should be noted that the nanomaterials above mentioned are also ideal building blocks for preparing the separation layer with tunable interlayer spacing, pore size and/or pore size distribution and abundant selective transport pathways [7]. Meanwhile, the additional functional groups and robust structure also endowed membrane with better anti‐fouling performance and stability. Thereinto, 2D nanomaterials exhibited uniform nanopores and stable physicochemical properties, which could effectively inhibit pore swelling and electrostatic screening effect at high salt concentrations. However, the large scale fabrication of regularly structured nanomaterials and their toxicity to organisms need to be studied in detail for practical applications.

In addition to the solute‐size‐related steric exclusion, Donnan effect induced by the electrostatic interaction between charged solutes and membrane surface, would also have significant effect on the separation performance [38]. For most commercial NF membranes with negative charges, the low rejection of cations limits their applications in removal of metal ions and cationic pigments. To improve the separation performance of cationic solutes, efforts have been made to the fabrication of membranes with (positively charged) amine moieties on the separation layers or surfaces [39]. However, most of biomolecules are negatively charged, which are easily adsorbed on membrane surface via electrostatic adsorption and cause membrane fouling, resulting in decline of permeate flux and selectivity. Recently, NF membrane with dually charged composite layer has attracted increasing attention. Such opposite charge pattern is conducive to reject both cations and anions as well as improve the anti‐fouling performance [40].

### 3.2 Chemical resistant NF membranes

**FIGURE 4:**
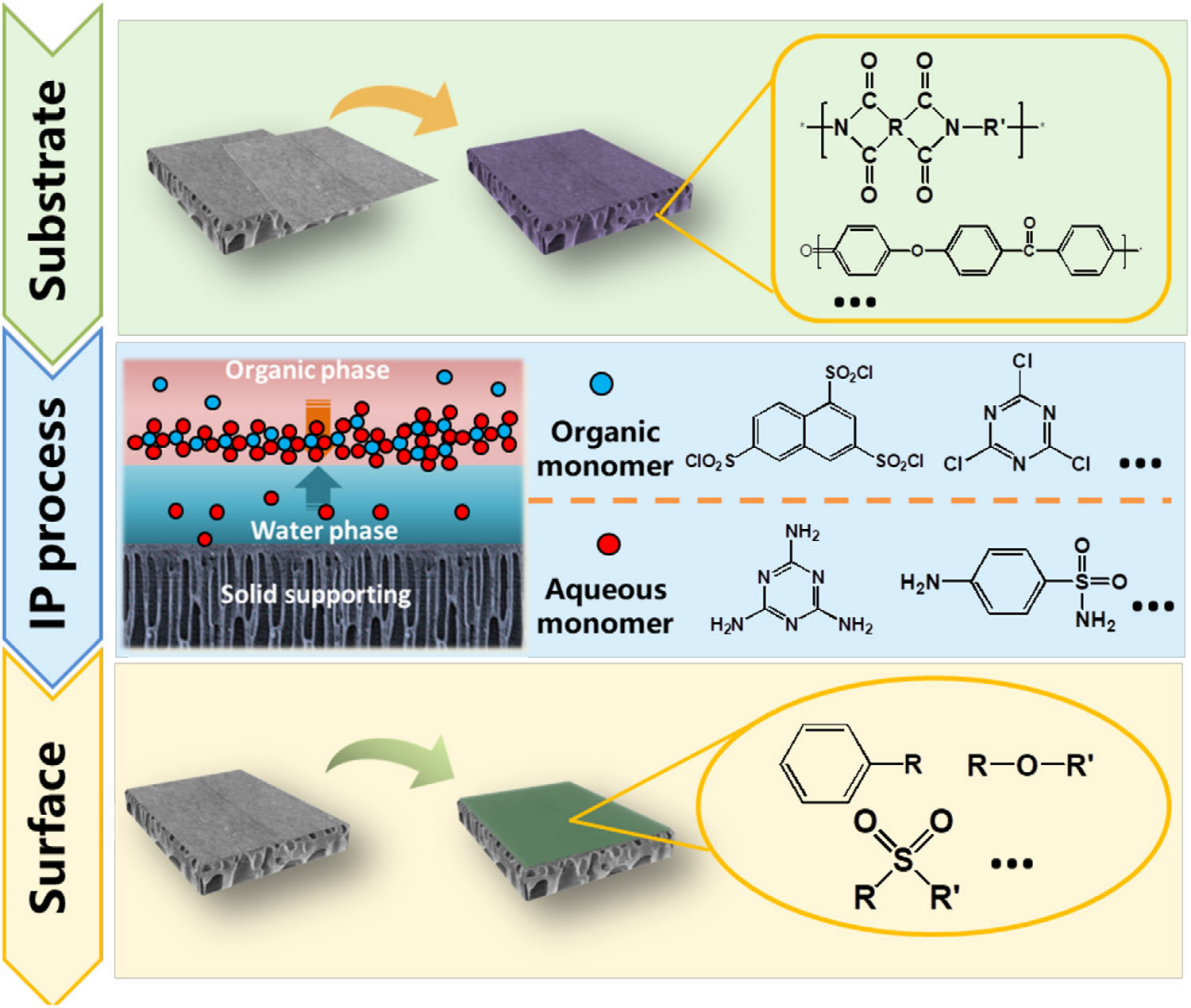
Overview of different methods for chemical stable NF membrane preparation

For some bio‐product manufacturing processes with massive acid/alkali and organic solvent consumption, the application of inorganic membranes (e.g. ceramic and metal) is an effective way to maintain the operation stability. However, the wide pore size distribution, low porosity, high‐cost and few commercial products of inorganic NF membranes limit their large‐scale application in bio‐separation [41]. Although many organic polymers have poor resistance to the acid/alkali and organic solvent, their chemical stability and anti‐swelling performance can be improved by introducing robust groups or monomers by various membrane modification techniques (Figure 4).

For acid resistance, chemical inert groups with strong conjugation effect such as phenyl rings, ether linkages, sulfone groups and heterocycles, can effectively improve the stability of chemical bonds [42]. Moreover, in order to obtain ideal permeability and selectivity, polar groups must be present on the polymer chains. Therefore, sulfonated aromatic polymers, such as sulfonated poly(ether ketone) and polysulphonamide are frequently investigated as ideal materials for preparing the acid resistant membrane [43, 44]. In addition, the rapid development of IP monomer provides more opportunities for the design of acid resistant PA skin‐layer. Although several commercial NF membranes (e.g. Duracid, Desal‐5‐DK, MPF‐34) have been used for high‐value product recovery from acidic wastewater, their high price and limited membrane choice impeded the large‐scale applications [44, 45]. Therefore, various monomers such as triazine‐amine, cyanuric chloride, sulfonamide and sulfonylchloride have been used to replace the traditional monomers to improve the stability [46, 47, 48, 49]. However, the acid stable groups (mostly electron‐withdrawing groups) with low nucleophilic would inevitably lead to a decline in monomer reactivity. To increase the cross‐linking degree of the separation layer, introducing co‐reactants and acid acceptor, as well as increasing the curing temperature have been employed [46, 50].

On the other hand, alkali resistant NF membranes are necessary for recovery of protein from chitosan production wastewater and regeneration of spent caustic washing water, and the high alkali resistance of the NF membrane can also increase the alkaline cleaning efficiency (using higher pH) and prolong life‐time of the membrane under frequent alkaline cleaning. However, only few commercially‐available NF membranes can endure the extreme pH conditions, include SelRO series, D‐series and HYDRACoRe series, and their price is quite high. Developing alternative NF membranes with outstanding alkali resistance and low‐price is imperative. Although some polymers with C–C bonds as the backbone have better hydrophilicity and excellent acid/base resistance, the poor membrane forming properties often require additional bulk or surface modifications to improve the cross‐linking degree of the separation layer [51]. For TFC membrane, besides the construction of compact sacrificial layer onto the surface, introducing bulky radical groups into the PA skin‐layer could suppress the alkali attacking by increasing the hindrance effect of OH^–^. For instance, Jiang et al. fabricated a pH stable TFC membrane using a mixture of PEI and piperazine (PIP) as the aqueous monomers, and cyanuric chloride (CC) as the organic monomer. The strong hindrance effect of chloride group in the molecules of CC could protect the amido group from the attack of OH^–^. Their results showed that the resulting PA layer structure and its separation performance could keep stable in alkali aqueous solution (pH 13) for 30 days [52].

Moreover, for the separation of biomolecules from organic solvent and the recovery of organic solvent during bio‐products manufacturing (e.g. food industry, pharmaceutical production), solvent‐resistant NF (SRNF) membrane is a promising solution. Unfortunately, as a weakly polar membrane material, the commonly used PES is not resistant to some polar solvents (i.e. ketones and chloroform) [27], and the PA separation layer would also seriously swell in organic solvent. Instead, polymers contain aromatic groups or imide bonds in the backbone such as polyimide (PI), polybenzimidazole (PBI) and poly(ether ether ketone) (PEEK) are preferentially used for preparing SRNF membrane [53]. Since the solubility of these polymers in organic solvent is not good and the resulting membrane has large pore size, further chemical crosslinking after membrane formation by some conventional materials is required to narrow the pore size of the SRNF membrane. Moreover, these materials could also be used as UF substrates for SRNF preparation. Introducing m‐phenylenediamine (MPD) into PI substrate, Yang et al. prepared a double layered SRNF membrane with PIP and trimesoyl chloride (TMC) by IP. Besides the defect free PA layer formed by PIP/MPD and TMC, the covalent interactions between MPD and PI substrate as well as hydrogen bonds in crosslinked PI provided a strong stability in organic solvents. After soaking in various solvents for 7 days, the rejection of Na_2_SO_4_ remained above 90% [54].

### 3.3 Thermal resistant NF membranes

In general, the suppression of microorganism growth during membrane filtration is a challenge, especially for the bio‐separation at large‐scale. Thermal resistant NF membrane can be operated at high temperatures, which not only limits microbial growth, but also reduces the feed viscosity lowering filtration resistance [55]. To achieve high permeability, stable solute rejection, less pore swelling and better anti‐fouling performance of NF membrane at high temperatures, researchers usually prepared thermal resistant TFC membranes via IP. Since the PA skin‐layer is attached to the porous substrate, severe swelling of the substrate at high temperatures would also cause the destruction of PA skin‐layer. Several polymers such as poly(phthalazine ether sulfone ketone) (PPESK), PVDF and PES were confirmed to withstand high temperature (60℃) and were suitable as the UF substrate for preparing TFC membrane [56]. Wei et al. used PPESK instead of polysulfone as the substrate to fabricate the thermal resistant NF membrane by IP, and the resultant membrane exhibited higher pigments rejection and less pores swelling at 80℃ compare with the pristine membrane [55]. Moreover, the thermal resistance of the TFC membrane with fully cross‐linked aromatic PA skin‐layer was substantially higher than that of the alicyclic aromatic and aliphatic aromatic structures. Therefore, monomers capable of forming highly cross‐linked PA layers such as 1,3,5‐triazine‐2,4,6‐triamine (melamine) and triaminopyrimidine are promising for the preparation of thermal resistant NF membranes [57, 58]. It is worth noting that these novel monomers usually suffer from low reactivity and/or low solubility, and thus adding highly reactive MPD/PIP is often required to increase the cross‐linking degree of the PA skin‐layer.

### 3.4 Anti‐fouling NF membranes

**FIGURE 5:**
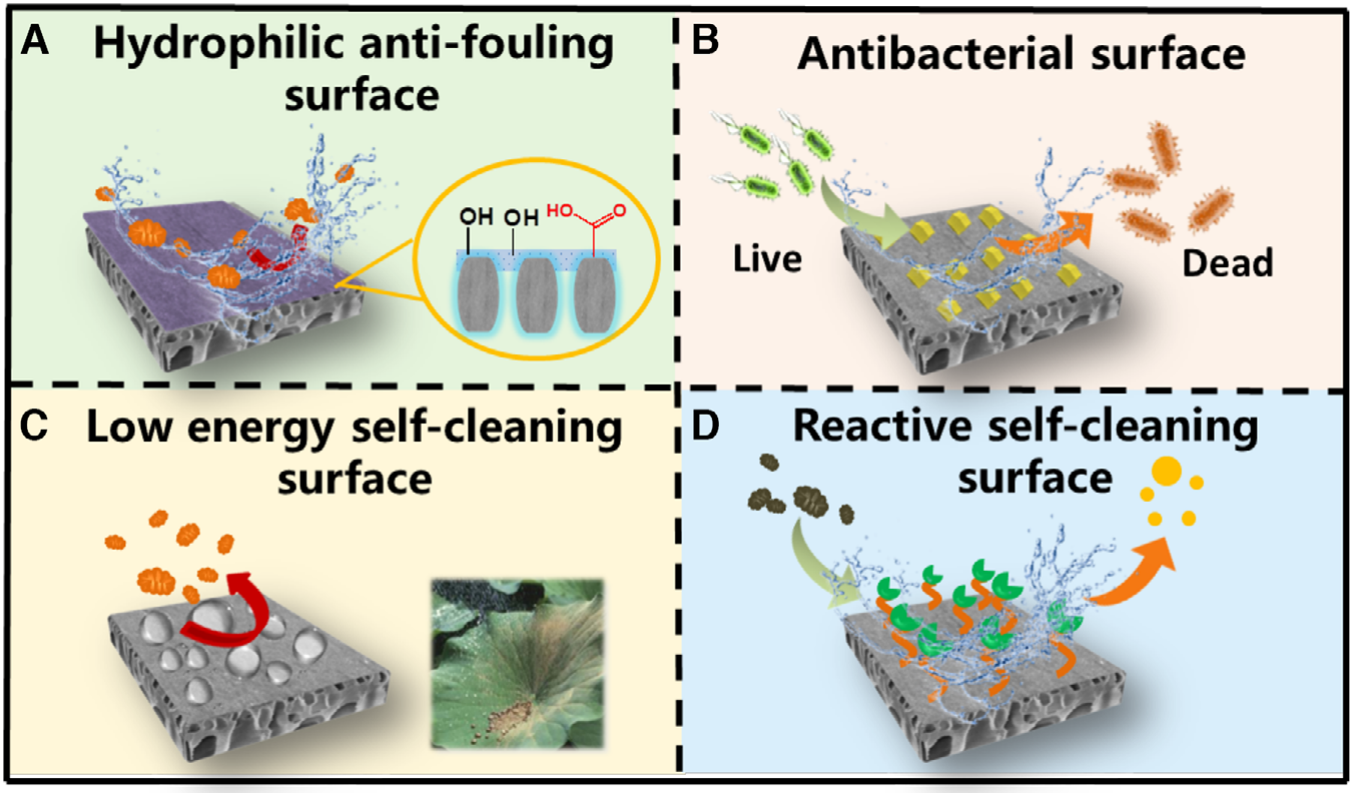
NF membranes with different surface properties for anti‐fouling improvement

Membrane fouling, especially the organic and microbial foulants (e.g. proteins, polysaccharide, cell debris) adsorbed in/on the membrane pores, is inevitable during the NF processes, leading to a sharp decline of permeate flux and a variation of separation selectivity [59, 60, 61]. From the perspective of materials innovation, surface properties are significant in determining the antifouling and separation performance of NF membranes, and thus most efforts have focused on the construction of antifouling surfaces, including hydrophilic anti‐fouling surface, antibacterial surface, low energy self‐cleaning surface and reactive self‐cleaning surface (Figure 5 and Table S2).

As shown in Figure 5A, surface hydrophilic modification is the most commonly used method, and hydrophilic groups on the membrane surface can interact with water molecules via hydrogen and ionic bonds to make them oriented on the surface and form a hydration layer; thus, reducing the probability of foulant adsorption. Sulfonation is a typical method to improve polymer (e.g. PES) hydrophilicity and biocompatibility, which can improve water permeability and inhibit biomolecules adhesion (especially for proteins) [62]. While for the PA membrane, diamine monomers with hydrophilic groups have been investigated for the preparation of anti‐fouling membrane. For instance, Ma et al. synthesized a zwitterionic amine monomer polyethylenimine‐sulfobetaine methacrylate (PEI‐g‐SBMA) and fabricated an electro‐neutral NF membrane by IP. The hydrophilic and zwitterionic molecules could reduce the electrostatic interactions between BSA and membrane surface [63]. Moreover, introducing additives such as hydrophilic/amphiphilic polymers and nanomaterials is also a facile and efficient strategy to inhibit the adsorption of foulants. In addition, the residual functional groups on the membrane surface (e.g. acyl chloride, amino groups) can be used as active site for post‐treatments, and hydrophilic polymers, nanomaterials and mussel‐inspired coating layers are easily grafted or deposited to modify the nascent PA membrane.

Anchoring antimicrobial agents on the membrane surface is also one of the priorities of the anti‐fouling membrane preparation (Figure 5B). Generally, antimicrobial agents can be divided into release bacteria‐killing type and contact bacteria‐killing type. It was reported that release bacteria‐killing chemicals such as antibiotics, phenols, and heavy metals could be introduced via various methods such as dip coating, in‐situ covalent functionalization, hydrogel trapping and direct deposition [64]. Alternatively, contact bacteria‐killing chemicals with long‐term durability such as quaternary amine compounds and phosphonium salts can be anchored on the membrane surface by chemical crosslinking. For instance, Wei et al. developed a multifunctional antibacterial surface by the layer‐by‐layer deposition of polyanion and quaternary ammonium salt, which killed more than 95% of attached bacteria (*Escherichia coli* and *Staphylococcus aureus*) by disrupting the negatively charged outer membranes of bacteria [65].

For low energy self‐cleaning surface, fluoropolymers and organosilicon polymers with abundant inert groups usually show superior anti‐fouling properties due to the inherent low surface free energy (Figure 5C). Actually, PTFE and PVDF are ideal material to fabricate microfiltration and UF membranes, but their poor membrane forming properties limit the application in the NF preparation. Therefore, doping fluoro groups into NF membrane materials was applied. Qian et al. synthesized perfluoro‐functionalized PEI and employed it as aqueous monomer in IP process. The covalently bonded perfluoro groups reduced surface free energy and the resultant membrane showed enhanced fouling resistance to BSA and humic acid, with a total flux decline ratios less than 10% [66].

Recently, reactive self‐cleaning surface such as biocatalytic and photocatalytic membranes has been constructed for antifouling enhancement (Figure 5D). Enzyme with various catalytic degradation roles can be immobilized in/on the membrane to degrade the specific pollutants which deposit or adsorb on the membrane, thereby decreasing fouling formation and flux decline. Dizge et al. fabricated a polyelectrolyte NF membrane for salt removal and the immobilized trypsin on the membrane outer layer for protein hydrolysis, and thus this biocatalytic NF membrane showed a minimal flux decline rate (8.45%) [67]. Moreover, since electron hole pair produced in photocatalytic reactions can further drive the degradation of foulants through redox reactions, photocatalyst materials (e.g. TiO_2_, TiO_2_‐polydopamine and N‐doped TiO_2_) with high stability, nontoxicity and biocompatibility are often integrated with membranes to control fouling accumulation [68]. Notably, for the real applications, the simplification of membrane preparation conditions, the dispersion of catalysts and their compatibility with the separation layer/substrates as well as the light accessibility in membrane module need to be further considered.

## 4 CONCLUDING REMARKS

A NF membrane with narrow pore size distribution, strong anti‐fouling surface as well as good thermal/chemical stability is desirable for the real applications in bio‐separation. Looking forward, there are many opportunities to improve this field further still. Membrane pore size regulation is necessary for highly selective separation and precise resource recovery. For the NF membranes prepared by phase inversion, bulk polymer functionalization and additive introduction are promising. While for the TFC membrane fabricated by IP, a substrate with high hydrophilicity and uniform pore size is quite important for obtaining defect free membrane with narrow pore size distribution. For harsh application environments, designing robust IP monomers with high reactivity is the key to endow the structure‐stable membrane with high cross‐linking degree (small and uniform pore size). Moreover, introducing chemical‐stable groups by surface modification strategies such as grafting, coating and layer‐by‐layer deposition may be used to improve the separation layer stability.

Besides the conventional materials, the emergence of innovate materials such as 2D nanosheets, microporous organic polymer, block copolymers and zwitterionic polymers are also ideal building blocks for the preparation of high‐performance molecular sieving separation layers [69]. Using simple pressure‐driven assembly method or in‐situ growth, these materials can form robust separation layers with ordered subnanometer sized pores, strong solvent and fouling resistance; thus, endowing the NF membranes with ultra‐high selectivity and structure stability.

Distinct from laboratory research, regular and frequent chemical membrane cleaning is required to restore the membrane performance, whereas the pore swelling caused by chemical cleaning reagents would accelerate the accumulation of foulants. Therefore, it is important to develop anti‐fouling and chemical‐resistant NF membrane as well as green cleaning agents to rapidly and mildly regenerate the membrane. Thereinto, the hyperbranched polymers, zwitterionic molecules and low surface energy polymers have shown great potential for the preparation of anti‐fouling surface due to their excellent ability of foulant repulsion. Moreover, thermal resistant NF membranes can be operated at high temperature to inhibit microbial growth, and it may be washed by hot water for disinfection; chlorine resistant NF membranes enable to be cleaned and sterilized by sodium hypochlorite, which both are conductive to control bacterial contamination. To further improve membrane cleaning efficiency, chemo‐enzymatic and photocatalytic reactions can be involved for off‐line fouling degradation or construction of reactive self‐cleaning surfaces (in‐situ cleaning).

In brief, materials innovation can solve most problems occurring during NF process, greatly reducing the whole cost of bio‐separation and accelerating the large‐scale applications of NF membrane in this emerging area.

## CONFLICT OF INTEREST

The authors have declared no conflict of interest.

## Supporting information

Supporting Information

## Data Availability

The data that support the findings of this study are available from the corresponding author upon reasonable request.
