# Supplementary material for: Nanofiltration membrane for bio‐separation: Process‐oriented materials innovation
Source: Eng Life Sci. 2021 Mar 20;21(6):405–16. doi: 10.1002/elsc.202000100 (PMC8182275; doi:10.1002/elsc.202000100)
Supplement: Supplementary file 1 — Supporting Information [file ELSC-21-405-s001.pdf]

Supporting information

Nanofiltration membrane for bio-separation: process-oriented materials innovation

Yang Cao[1,2]

Guoqiang Chen [1]

Yinhua Wan[1,2]

Jianquan Luo[1,2*]

[1] State Key Laboratory of Biochemical Engineering, Institute of Process Engineering, Chinese Academy of Sciences, Beijing 100190, China

[2] School of Chemical Engineering, University of Chinese Academy of Sciences, Beijing 100049, PR China

Table S1 Bio-separation performances of various NF membranes prepared with innovate membrane materials

Membrane type	Permeance ($Lm^{-2}h^{-1}bar^{-1}$)	Separation performance	Application	Refs
NTR7450	5.3	Molasses decolorization rate: >90%	Bio-product decolorization	[1]
HNTs-SO_3H/PES	18.7	Reactive black rejection: > 90%; Na_2SO_4 rejection: <10%	Bio-product decolorization	[2]
TiO_2-HDMI modified PES membrane	15.3	Congo red rejection: 97.4%; Methyl blue rejection: 99.1%; Na_2SO_4 rejection: 6%	Bio-product decolorization	[3]
PEI-PI	37.4	Jansu green B rejection: 98.7%;	Bio-product decolorization	[4]
ZTFCMs	8.4	Erythromycin rejection: 96.5%; NaCl rejection: 14.4%	Biomolecules separation	[5]
PEI-g-SBMA/TMC	13.2	Orange GII rejection:90.6%; Na_2SO_4 rejection:50.4%; NaCl rejecton:7.1%	Bio-product decolorization	[6]
PA-PSFn	14.8	Norfloxacin rejection: >90%; NaCl rejection: <b20%	Biomolecules separation	[7]
N-hexane post-treated PA membrane	16.7	Clindamycin rejection: 95%; NaCl rejection: 19%	Bio-product desalination	[8]
Ethanol post-treated PA membrane	19.3	Clindamycin rejection: 92%; NaCl rejection: 12%	Bio-product desalination	[8]
HGMX	89.6	Rhodamine B rejection: >97%;, Methyl blue rejection: >97%	Bio-product decolorization	[9]

Abbreviations: Halloysite nanotubes (HNTs); 1, 6-hexamethylene diisocyanate (HDMI); polyimide-Extem (PI); zwitterionic polyamide thin film composite nanofiltration membranes (ZTFCMs); Sulfobetaine methacrylate (SBMA); Polysulfone (PSF); GO-based NF membrane intercalated with TiO_2 nanoparticles (HGMX).

Table S2 Fouling bahavior of various membranes and the corresponding anti-fouling mechanisms

Membrane type	Anti-fouling performance	Regulation mechanisms	Refs
PEG-4arm-NH_2/TMC	• Flux recovery ratio >90% after fouling with BSA	• PEG enhances the surface hydrophilicity	[10]
PEI-g-SBMA/TMC	• Number of *Escherichia coli* adhered on to surface is significantly lower than the control membrane	• Zwitterionic monomer provides a hydrophilic and electroneutral membrane surface	[6]
ZA-modified PA membrane	• Flux recovery ratio >99% after fouling with BSA	• Groups of ZA improve the hydrophilicity of membrane<br>• The self-restrictive diffusion effect of PIP/ZA obtained from the reaction of PIP and ZA result in smooth membrane surface	[11]
P(AA-co-Ada)/PAH	• *Escherichia coli* and *Staphylococcus aureus* killing ratio >95%	• Quaternary ammonium salt groups on the surface inhibit the growth of bacteria	[12]
FPA/PES	• Total flux decline ratio is 9.9% and 4.9% after fouling with BSA and HA, respectively	• The covalently bonded perfluoro groups effectively lower the surface free energy of the membrane	[13]
PDADMAC-PSS	• The flux almost keeps constant after fouling with BSA for 24 h	• Biocatalytic activity removes adsorbed proteins from modified membrane surface.	[14]

Abbreviations: Polyethylene glycol (PEG); bovine serum albumin (BSA); zoledronic acid (ZA); piperazine (PIP); Poly(acrylic acid-co-1-adamantan-1-ylmethyl acrylate)/Poly(allylamine hydrochloride) (P(AA-co-Ada)/PAH); fluorinated polyamide (FPA); poly(diallyl dimethyl ammonium chloride) (PDADMAC); poly(acrylic acid) (PAA).

References

[1] Luo, J., Guo, S., Qiang, X., Hang, X., et al., Sustainable utilization of cane molasses by an integrated separation process: Interplay between adsorption and nanofiltration. *Sep. Purif. Technol.* 2019, *219,* 16-24.

[2] Wang, Y., Zhu, J., Dong, G., Zhang, Y., et al., Sulfonated halloysite nanotubes/polyethersulfone nanocomposite membrane for efficient dye purification. *Sep. Purif. Technol.* 2015, *150,* 243-251.

[3] Zhang, L., Guan, H., Zhang, N., Jiang, B., et al., A loose NF membrane by grafting TiO2-HMDI nanoparticles on PES/β-CD substrate for dye/salt separation. *Sep. Purif. Technol.* 2019, *218,* 8-19.

[4] Xia, Q.-C., Yang, W.-J., Fan, F., Ji, M., et al., Encapsulated Polyethyleneimine Enables Synchronous Nanostructure Construction and In Situ Functionalization of Nanofiltration Membranes. *Nano Lett.* 2020, *20,* 8185-8192.

[5] Weng, X.-D., Ji, Y.-L., Ma, R., Zhao, F.-Y., et al., Superhydrophilic and antibacterial zwitterionic polyamide nanofiltration membranes for antibiotics separation. *J. Membr. Sci.* 2016, *510,* 122-130.

[6] Ma, T., Su, Y., Li, Y., Zhang, R., et al., Fabrication of electro-neutral nanofiltration membranes at neutral pH with antifouling surface via interfacial polymerization from a novel zwitterionic amine monomer. *J. Membr. Sci.* 2016, *503,* 101-109.

[7] Yang, Z., Wang, F., Guo, H., Peng, L.E., et al., Mechanistic Insights into the Role of Polydopamine Interlayer toward Improved Separation Performance of Polyamide Nanofiltration Membranes. *Environ. Sci. Technol.* 2020, *54,* 11611-11621.

[8] Guo, S., Chen, X., Wan, Y., Feng, S., et al., Custom-Tailoring Loose Nanofiltration Membrane for Precise Biomolecule Fractionation: New Insight into Post-Treatment Mechanisms. *ACS Appl. Mater. Interfaces*. 2020, *12,* 13327-13337.

[9] Han, R. and Wu, P., High-performance graphene oxide nanofiltration membrane with continuous nanochannels prepared by the in situ oxidation of MXene. *J. Mater. Chem. A*. 2019, *7,* 6475-6481.

[10] Cheng, X.Q., Liu, Y., Guo, Z. and Shao, L., Nanofiltration membrane achieving dual resistance to fouling and chlorine for "green" separation of antibiotics. *J. Membr. Sci.* 2015, *493,* 156-166.

[11] Cao, X.-L., Zhou, F.-Y., Cai, J., Zhao, Y., et al., High-permeability and anti-fouling nanofiltration membranes decorated by asymmetric organic phosphate. *J. Membr. Sci.* 2021, *617,* 118667.

[12] Wei, T., Zhan, W., Cao, L., Hu, C., et al., Multifunctional and Regenerable Antibacterial Surfaces Fabricated by a Universal Strategy. *ACS Appl. Mater. Interfaces*. 2016, *8,* 30048-30057.

[13] Qian, Y., Wu, H., Sun, S.-P. and Xing, W., Perfluoro-functionalized polyethyleneimine that enhances antifouling property of nanofiltration membranes. *J. Membr. Sci.* 2020, *611,* 118286.

[14] Dizge, N., Epsztein, R., Cheng, W., Porter, C.J., et al., Biocatalytic and salt selective multilayer polyelectrolyte nanofiltration membrane. *J. Membr. Sci.* 2018, *549,* 357-365.
